# Lichen Secondary Metabolites in *Flavocetraria cucullata* Exhibit Anti-Cancer Effects on Human Cancer Cells through the Induction of Apoptosis and Suppression of Tumorigenic Potentials

**DOI:** 10.1371/journal.pone.0111575

**Published:** 2014-10-31

**Authors:** Thanh Thi Nguyen, Somy Yoon, Yi Yang, Ho-Bin Lee, Soonok Oh, Min-Hye Jeong, Jong-Jin Kim, Sung-Tae Yee, Florin Crişan, Cheol Moon, Kwang Youl Lee, Kyung Keun Kim, Jae-Seoun Hur, Hangun Kim

**Affiliations:** 1 Korean Lichen Research Institute, Sunchon National University, Sunchon, Republic of Korea; 2 Faculty of Natural Science and Technology, Tay Nguyen University, Buon Ma Thuot, Vietnam; 3 Medical Research Center for Gene Regulation, Chonnam National University Medical School, Gwangju, Republic of Korea; 4 College of Pharmacy and Research Institute of Life and Pharmaceutical Sciences, Sunchon National University, Sunchon, Republic of Korea; 5 Department of Taxonomy and Ecology, Faculty of Biology and Geology, Babes′-Bolyai University, Cluj-Napoca, Romania; 6 College of Pharmacy, Chonnam National University, Gwangju, Korea; Michigan State University, United States of America

## Abstract

Lichens are symbiotic organisms which produce distinct secondary metabolic products. In the present study, we tested the cytotoxic activity of 17 lichen species against several human cancer cells and further investigated the molecular mechanisms underlying their anti-cancer activity. We found that among 17 lichens species, *F. cucullata* exhibited the most potent cytotoxicity in several human cancer cells. High performance liquid chromatography analysis revealed that the acetone extract of *F. cucullata* contains usnic acid, salazinic acid, Squamatic acid, Baeomycesic acid, d-protolichesterinic acid, and lichesterinic acid as subcomponents. MTT assay showed that cancer cell lines were more vulnerable to the cytotoxic effects of the extract than non-cancer cell lines. Furthermore, among the identified subcomponents, usnic acid treatment had a similar cytotoxic effect on cancer cell lines but with lower potency than the extract. At a lethal dose, treatment with the extract or with usnic acid greatly increased the apoptotic cell population and specifically activated the apoptotic signaling pathway; however, using sub-lethal doses, extract and usnic acid treatment decreased cancer cell motility and inhibited *in* *vitro* and *in* *vivo* tumorigenic potentials. In these cells, we observed significantly reduced levels of epithelial-mesenchymal transition (EMT) markers and phosphor-Akt, while phosphor-c-Jun and phosphor-ERK1/2 levels were only marginally affected. Overall, the anti-cancer activity of the extract is more potent than that of usnic acid alone. Taken together, *F. cucullata* and its subcomponent, usnic acid together with additional component, exert anti-cancer effects on human cancer cells through the induction of apoptosis and the inhibition of EMT.

## Introduction

Cancer is a major cause of death worldwide. As a group, cancers account for approximately 13% of all deaths each year with the most common being lung cancer (1.37 million deaths), stomach cancer (736,000 deaths), liver cancer (695,000 deaths), colorectal cancer (608,000 deaths), and breast cancer (458,000 deaths) [1]. Invasive cancer is the leading cause of death in the developed world and the second leading cause of death in the developing world [2], so for these reasons, various cancer therapies have been developed, including a wide range of anti-cancer agents with known cytotoxic effects on cancer cells.

Lichens are symbiotic organisms, usually composed of a fungal partner (mycobiont) and one or more photosynthetic partners (photobiont), which is most often either a green alga or a cyanobacterium [3]. Although the dual nature of most lichens is now widely recognized, it is less commonly known that some lichens are symbioses involving three (tripartite lichens) or more partners. In general, lichens exist as discrete thalli and are implicitly treated as individuals in many studies, even though they may be a symbiotic entity involving species from three kingdoms. From a genetic and evolutionary perspective, lichens cannot be regarded as individuals but rather as composites, and this has major implications for many areas of investigation such as development and reproduction.

Many lichen secondary products are unpalatable and may serve as defensive compounds against herbivores as well as decomposers. For this reason, these secondary products are frequently used by the pharmaceutical industry as antibacterial and antiviral compounds [4], [5]. In addition, lichens and their secondary metabolites have long been studied for anti-cancer therapy [6]–[15]. In the present study, we tested the cytotoxic activity of 17 lichen species collected from the Romanian Carpathian mountains against several human cancer cells and further investigated the molecular mechanisms underlying their anti-cancer activity to identify potential compounds for novel anti-cancer agents.

## Materials and Methods

### Preparation of lichen extracts

Thalli of *F. cucullata* were collected from Romania in 2011 during the field trip in the National Park Călimani and the Natural Park Bucegi organized by Dr. Crişan at Babeş-Bolyai University, Cluj-Napoca, Romania. The permit to collect lichen specimens from those locations was issued by the Administration of the National Park Călimani and the Administration of the Natural Park Bucegi, with the approval of the Commission for Protection of Natural Monuments (Romanian Academy). The field studies did not involve any endangered or protected species. The duplicates were deposited into the Korean Lichen Research Institute (KoLRI), Sunchon National University, Korea. Finely dried ground thalli of the lichen (150 g) were extracted using acetone in a Soxhlet extractor. The extracts were filtered and then concentrated under reduced pressure in a rotary evaporator. The dry extracts were stored at −25°C until further use. The extracts were dissolved in dimethylsulfoxide (DMSO) for all experiments.

### High performance liquid chromatography (HPLC) analysis of lichen materials

Dry lichen extracts were redissolved in 2 mL of acetone and then subjected to HPLC (SHIMADZU, LC-20A). HPLC analyses were carried out on YMC-Pack ODS-A (150×3.9 mm I.D.) reversed-phase column fully endcapped C18 material (particle size, 5 µm; pore size, 12 nm). Elution was performed at a flow rate of 1 mL/min under the following conditions: column temp, 40°C; solvent system, methanol: water: phosphoric acid (80∶20∶1, v/v/v) before subsequent injection. The analysis was monitored by a photodiode array detector (SPD-M20A) with a range of 190–800 nm during the entire HPLC run. Observed peaks were scanned between 190 and 400 nm. The sample injection volume was 10 µL. The standards used were obtained from the following sources: salazinic acid (t_R_ = 2.27±0.2 min) isolated from lichen *Lobaria pulmonaria,* usnic acid (t_R_ = 11.3±0.3 min) from *Usnea longissima* and protolichesterinic acid (t_R_ = 22.3±0.2 min) and lichesterinic acid (t_R_ = 26.5±0.2 min) from lichen *Cetraria islandica*.

### Cell culture

The human cancer cell lines HT29 (colon cancer), AGS (gastric cancer), A549 (lung cancer), and CWR22Rv-1 (prostate cancer) were maintained in Roswell Park Memorial Institute (RPMI) 1640 medium (Gen Depot, USA) supplemented with 10% fetal bovine serum (FBS) (Gen Depot, USA) and 1% penicillin and streptomycin (RPMI complete medium) (Gen Depot, USA). HaCaT (human keratinocyte), NIH 3T3 (mouse embryonic fibroblast cells), HEK293T (human embryonic kidney) cells, RIE (rat intestinal epithelial) cells, and Madin-Darby canine kidney (MDCK) cells were maintained in Dulbecco’s Modified Eagle Medium (DMEM) (Gen Depot, USA) supplemented with 10% FBS and 1% penicillin and streptomycin. Cells were cultured in 5% CO_2_ in a humidified atmosphere at 37°C. Cell lines were purchased from the Korean Cell Line Bank (http://cellbank.snu.ac.kr), Korea.

### MTT assay

Lichen extracts were dissolved in DMSO (Sigma-Aldrich, St. Louis, USA) and serially diluted with DMEM or RPMI 1640 to obtain concentrations of 6.125, 12.5, 25, 50, 100 µg/mL. Cells (2×10^4^ cells/well) were seeded on a 96-well plate, grown overnight, and then treated with the acetone extract or main compounds of *F. cucullata* at concentrations of 100 µg/mL or µM to 10 µg/mL or µM for 48 hr. Once treatment was completed, cultures were supplemented with MTT. After incubation with MTT at 37°C, cells were lysed with lysis buffer containing 50% DMSO and 20% SDS, and absorbance was measured at 570 nm using a microplate reader (VERSAmax, Molecular Devices, Minnesota, USA). The percentage of viable cells was calculated using the following formula: percentage cell viability = (optical density (OD) of the experiment samples/OD of the control)×100. IC_50_ values were calculated using the Statistical Package for Social Science (SPSS) software.

### Fluorescence microscopy of apoptotic morphology

Cells were cultured on chamber slides at a density of 4×10^5^ cells/well and allowed to attach overnight, followed by treatment with the *F. cucullata* acetone extract or usnic acid for 24 hr or 48 hr. Cells were washed with phosphate-buffered saline (PBS) and incubated with Annexin V FITC labeling solution for 15 min. Then, cells were washed twice in PBS and analyzed using a Nikon Eclipse 400 (Nikon Instech Co., Ltd., Kawasaki, Japan) fluorescent microscope.

Cells were cultured as described above. After 24 hr or 48 hr treatment with the *F. cucullata* acetone extract or usnic acid, cells were washed with PBS three times and fixed in 4% paraformaldehyde at room temperature, then incubated in 0.1% Triton x-100 (Sigma-Aldrich, St. Louis, USA) for 30 min. Subsequently, the samples were stained with Hoechst 33257 (Sigma-Aldrich, St. Louis, USA) at room temperature after washing three times in fixative solution in PBS. The cells were washed twice in PBS and mounted onto a glass slide. The slides were analyzed using a Nikon Eclipse 400 (Nikon Instech Co., Ltd., Kawasaki, Japan) fluorescent microscope and evaluated as the percentage of cells with condensed or fragmented nuclei from a total number of 300 cells.

### Flow cytometry assay for cell cycle

After 24 hr of incubation, untreated and acetone extract- or usnic acid-treated MDCK, HEK293T, HT29, AGS, A549, and CWR22Rv-1 cells were trypsinized. Then, an RNase inhibitor was added and incubated for 15 min at room temperature. Finally, the samples were centrifuged to remove the supernatant, and pellet cells were diluted in 100 µL of propidium iodide (Sigma-Aldrich, St. Louis, USA) and incubated for another 30 min at 4°C. Flow cytometry was performed with a FACS Caliber (BD Biosciences, San Diego, USA).

### Wound-healing assay

AGS and A549 cells were plated at a density of 2.5×10^5^ cells/well on 6-well tissue culture plates (Corning, New York, USA) and grown overnight to confluence. Monolayer cells were scratched with a pipette tip to create a wound. The cells were then washed twice with serum-free RPMI 1640 to remove floating cells and incubated in medium with 5 µg/mL of the *F. cucullata* extract or 10 µM usnic acid. Photographs of cells were taken at 0, 24, 48, and 72 hr after wounding to measure the width of the wound. The distance migrated by the cells was calculated as the difference between the edges of the wound at time point 1 and at time point 2. For each cell line, an average of eight wound assays was taken to determine the average rate of migration at a given concentration of acetone extract or usnic acid. Experiments were repeated at least three times.

### Invasion assay

Tumor cell invasion was analyzed using a transwell chamber (Corning Coster, Corning, NY, USA) assay with a topchamber of 8 µm pore-size polycarbonate membrane coated with 1% gelatin. AGS and A549 cells were plated at 2.5×10^5^ cells/well in culture medium containing 0.2% bovine serum albumin (BSA) in the upper compartment of the chamber. The lower compartment was filled with culture medium containing 0.2% BSA and 1 µg/mL fibronectin as a chemo-attractant. Cells were cultured in the absence or presence of 5 µg/mL acetone extract or 10 µM usnic acid for 24 hr. The upper chambers were fixed and stained with Diff Quick kit (Sysmex, Kobe, Japan). The invaded cells were analyzed under a light microscope in five randomly selected fields. Each experiment was performed in triplicate. The results are expressed as the mean number of cells migrating per high-power field.

### Clonogenic assay

A549 and AGS cells were washed, trypsinized, and resuspended in RPMI 1640. Cells (500 cells/well) were seeded on 6-well plates in 2.5 mL RPMI 1640 medium per well and were incubated for attachment. Subsequent to 48 hr treatment, media containing the acetone extract or usnic acid was replaced with fresh medium for 12 days. Colonies were fixed in 4% paraformaldehyde, stained with 0.5% crystal violet, and counted under a stereomicroscope. The plating efficiency (PE) of untreated cells and the survival fraction (SF) of treated cells were then determined (n = 3) [16].

### Soft agar colony-formation assay

AGS (1×10^4^) and A549 (1×10^4^) cells were suspended in 1.5 mL of soft agar (0.35% agarose in RPMI complete medium), plated onto 1.5 mL of solidified agar (0.6% agarose in RPMI complete medium) in 6-well plates and cultured for 3 weeks. Cells were fed two times per week with cell culture media containing the acetone extract (1 µg/mL or 5 µg/mL), usnic acid (5 µM or 10 µM), lichesterinic acid (10 µM), or DMSO (0.01%). Pixel intensity of colony area was measured by the IMT iSolution software (IMT i-Solution Inc., Northampton, NJ, USA) in randomly selected microscope fields in each plate. To measure the percent area of colony, pixel amount of colony area were normalized to pixel×pixel square. Data represent the mean of three experiments.

### Detection of proteins in cell lysates

Cells treated with various concentrations of the acetone extract or subcomponents for 48 hr were harvested and analyzed by western blot as previously described [17]. The antibodies used were purchased from Cell signaling Technology (PARP, caspase-3, Bax, Bcl_xL, α-tubulin), and BD Biosciences (E-cadherin). All results are representative from at least three independent experiments. To analyze the level of phosphoproteins, bead-based multiplex assay (Bio-Plex phosphoprotein assay, Bio-Rad, Hercules, CA, USA) was performed according to the manufacturer’s instructions [18], [19]. Briefly, this assay measured multiple phosphoprotein signals from a single lysate [20].

### qRT-PCR analysis

Total RNA (1 µg) from each group of treated cells was converted to cDNA using a M-MLV reverse Transcriptase kit (Invitrogen, Carlsbad, CA, USA) and SYBR green (Enzynomics, Seoul, Korea). The primers used for real-time PCR were E-cadherin (forward) 5′-cagaaagttttccaccaaag-3′ and (reverse) 5′-aaatgtgagcaattctgctt-3′; N-cadherin (forward) 5′-ctcctatgagtggaacaggaacg-3′ and (reverse) 5′-ttggatcaatgtcataatcaagtgctgta-3′; Snail (forward) 5′-gaggcggtggcagactag-3′ and (reverse) 5′-gacacatcggtcagaccag-3′; Twist (forward) 5′-cgggagtccgcagtctta-3′ and (reverse) 5′-tgaatcttgctcagcttgtc-3′; GAPDH (forward) 5′-atcaccatcttccaggagcga-3′ and (reverse) 5′-agttgtcatggatgaccttggc-3′. qRT-PCR reaction and analysis were performed using CFX (Bio-Rad, Hercules, CA, USA).

### In vivo tumorigenicity assay

The experimental protocol was approved by the Chonnam National University Medical School Research Institutional Animal Care & Use Committee. Maintenance of animals and all in vivo experiments were performed according to the Guiding Principles in the Care and Use of Animals (DHEW publication, NIH 80-23). A549 cells were prepared in RPMI 1640 medium (1×10^6^ cells/mouse) and suspended cells were pretreated with one-tenth of lethal concentration of *F. cucullata* acetone extract, usnic acid, lichesterinic acid, or DMSO (Vehicle) just before injection. Cells were injected subcutaneously into the flank region of Balb/c nude mouse and tumor was measured after two weeks.

### Statistical analysis

All experiments were assayed in triplicates (n = 3). Data were expressed as means ± standard deviation. All statistical analyses were performed using the SPSS version 17. Treatment effects were determined using one-way ANOVA post-hoc analysis. A p-value<0.05 was considered significant unless indicated otherwise.

## Results

### *Flavocetraria cucullata* acetone extract has potent cytotoxic effects on cancer cells

**Figure 1:**
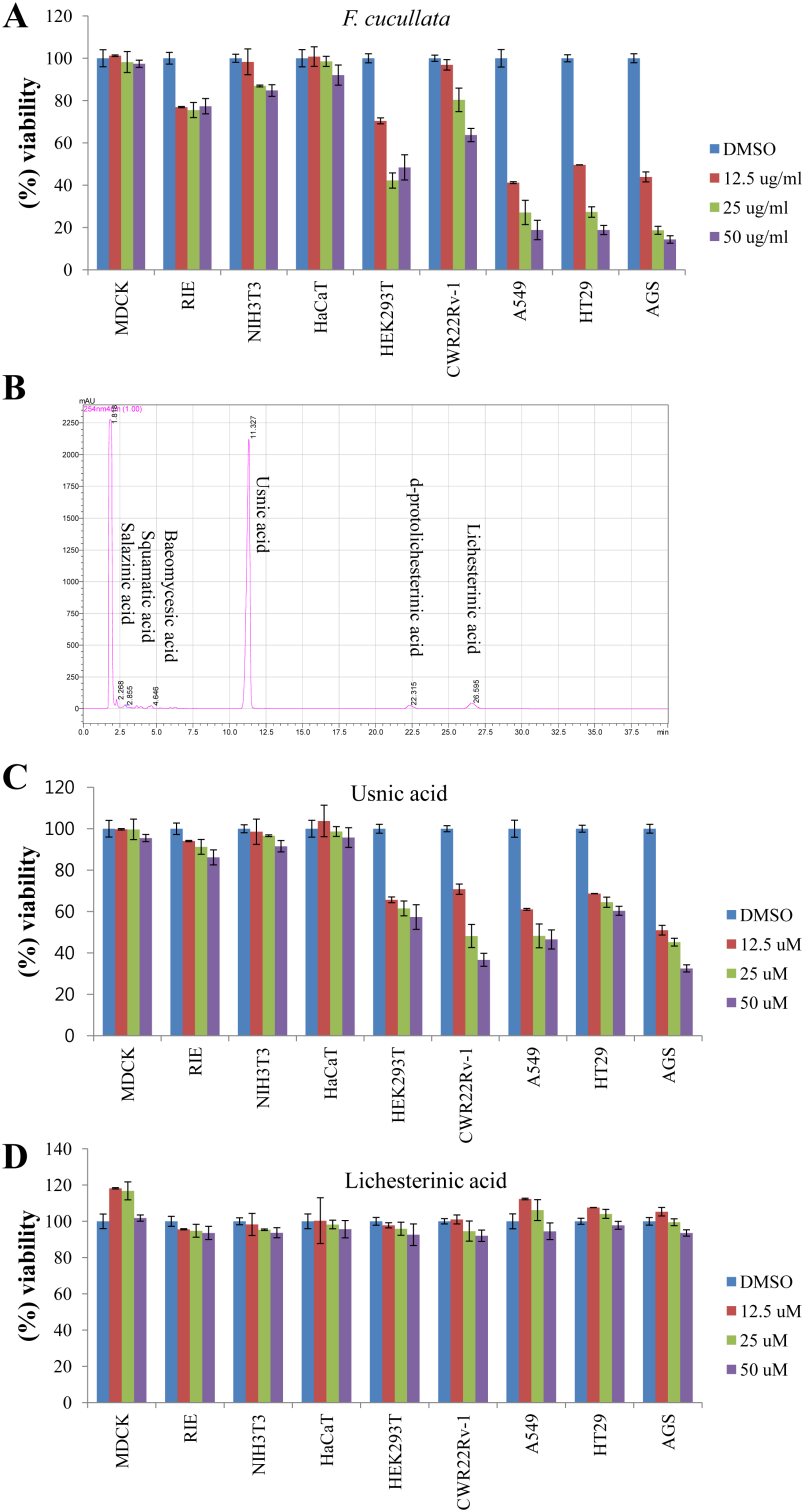
Cytotoxic effects of the acetone extract of *F. cucullata* and its main component, usnic acid, on human cancer cells. (A) Percent viability of cells treated with the acetone extract of *F. cucullata*. Cells were treated with the *F. cucullata* extract in a concentration ranging from 10–50 µg/mL for 48 hr, and cell viability was measured by an MTT assay. (B) High performance liquid chromatography chromatograms of the *F. cucullata* extract. The identity of each subcomponent is noted on the corresponding peak. (C–D) The percent viability of cells treated with either usnic acid (C) or lichesterinic acid (D). Cells were treated with the indicated subcomponent of *F. cucullata* in a concentration ranging from 12.5–50 µM for 48 hr, and cell viability was measured by an MTT assay. Data represent means ± S.E.M. (standard error of the mean), n = 3.

To identify the cytotoxic substance from Romanian lichens, we measured the IC_50_ values of acetone extracts from 17 lichen species on HT29 (colorectal cancer cells) and AGS (gastric cancer cells) cells. Among the lichen species, *F. cucullata* exerted the most potent cytotoxic effects on both HT29 (IC_50_ = 10.9 µg/mL) and AGS (IC_50_ = 11.6 µg/mL) compared to other lichen species (IC_50_ values ranged around 25–100 µg/mL, Table S1 in File S1). To verify whether the cytotoxicity of the *F. cucullata* extract was specific to cancer cells, we conducted further tests on additional cancer cells including A549 (lung cancer cells) and CWR22Rv-1 (prostate cancer cells) cells and on non-cancer cells including MDCK (Madin-Darby canine kidney) and RIE (rat intestinal epithelial cell), NIH 3T3 (mouse embryonic fibroblast), HaCaT (human keratinocyte) cells. The results showed that the *F. cucullata* acetone extract specifically affected the viability of cancer cells while non-cancer cells were not severely damaged (Fig. 1A).

To identify the subcomponents of *F. cucullata*, HPLC was performed on the acetone extract of *F. cucullata*; the resulting chromatograms and mass spectrometric analyses of each peak are presented in Figure 1B and Table S2 in File S1. As shown in Figure 1B, salazinic acid (Tr = 2.268 min), squamatic acid (Tr = 2.855), baeomycesic acid (Tr = 4.646), usnic acid (Tr = 11.327), d-protolichesterinic acid (Tr = 22.315), and lichesterinic acid (Tr = 26.595) were detected in the acetone extract of *F. cucullata*. Among the subcomponents, usnic acid had the highest peak (% intensity = 91.49±0.0025), while d-protolichesterinic acid and lichesterinic acid showed lower peaks (% intensity = 2.27±0.1, 2.22±0.1, respectively) (Table S2 in File S1). After identifying the lichen subcomponents, two main metabolites, usnic acid (Sigma-Aldrich, St. Louis, USA) and lichesterinic acid (isolated from *F. cucullata* extract which share their chemical structure with d-protolichesterinic acid except one unsaturated carbon bond) were used for further experiments. To determine which subcomponent was responsible for the cytotoxicity of lichen, we measured the cytotoxicity of usnic acid and lichesterinic acid and found that usnic acid showed similar cytotoxic effects in non-cancer and cancer cells while lichesterinic acid exhibited no apparent cytotoxic effect on any of the tested cells (Fig. 1C and 1D). In Table S3 in File S1, the IC_50_ values of acetone extract, usnic acid, and lichesterinic acid in various cells are presented. Interestingly, given the molecular weight (MW = 344) and % intensity of usnic acid in the acetone extract of *F. cucullata*, IC_50_ values of usnic acid in HEK293T, HT29, and A549 cells were likely higher than those of calculated usnic acid concentration in the extract. These results suggest that unidentified subcomponents of the *F. cucullata* extract likely potentiate the cytotoxicity of usnic acid. However, it is worth noting that IC_50_ values of usnic acid, especially in non-cancer cells such as MDCK, RIE, NIH 3T3, and HaCaT cells, were much lower than those of usnic acid concentration in the *F. cucullata* extract, suggesting that there may be a resistant mechanism underlying the cytotoxicity of usnic acid in cells or that other subcomponent(s) can lessen the cytotoxicity of usnic acid to the cells. Taken together, these results suggest that the acetone extract of *F. cucullata* has selective cytotoxicity to cancer cells and that usnic acid may act as a major effector of these effects.

### Lethal concentrations *of Flavocetraria cucullata* and usnic acid induce apoptosis of cancer cells

**Figure 2:**
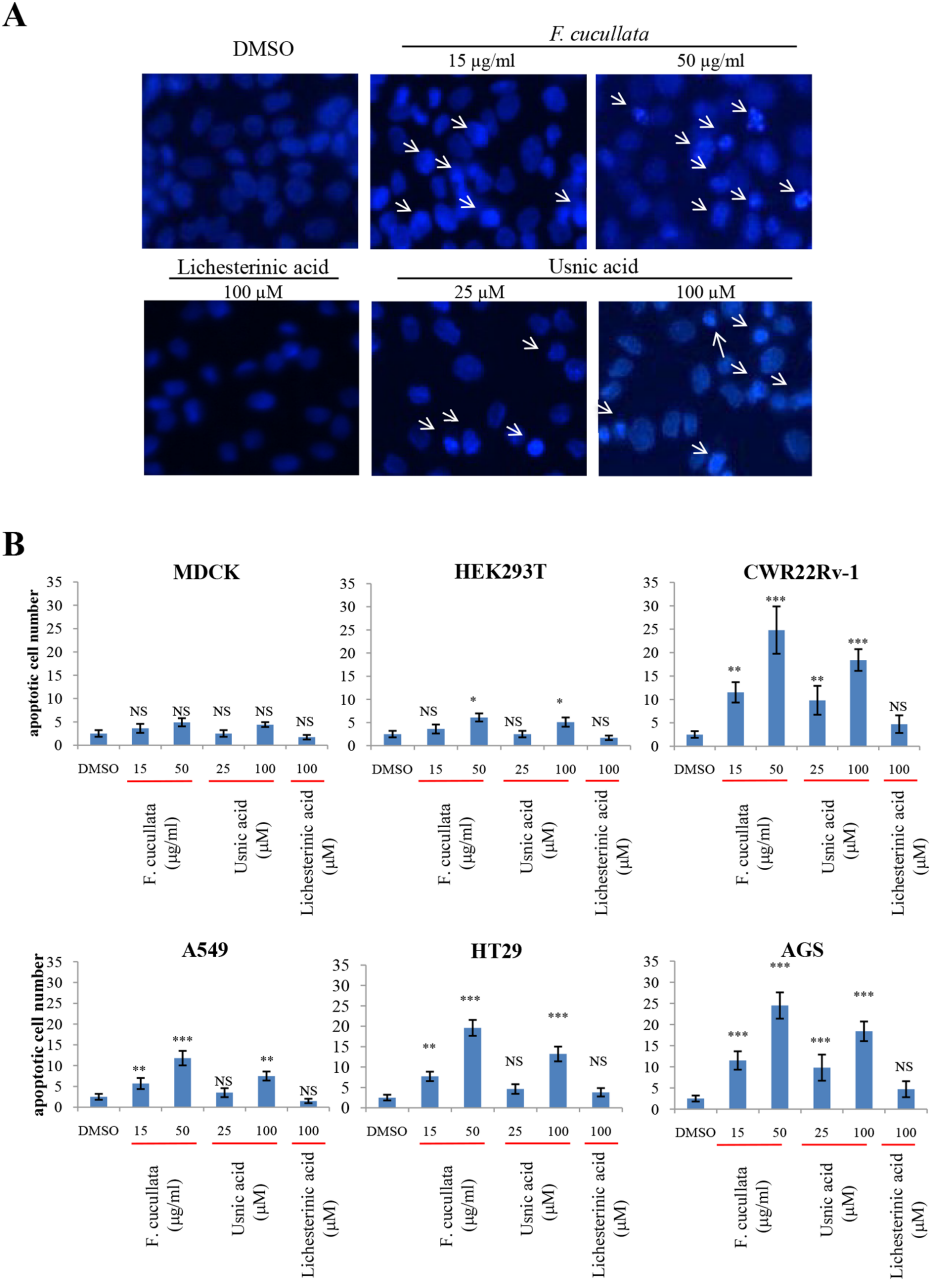
Induction of nuclear condensation of human cancer cells by the acetone extract of *F. cucullata* and its main component, usnic acid in lethal concentrations. (A) Hoechst 33258 staining of AGS (human gastric cancer cell line) cells treated with the *F. cucullata* extract or its subcomponents, usnic acid and lichesterinic acid. Arrows indicate cells showing condensed or fragmented nuclear morphology. Representative images are shown from three independent experiments. (B) Quantificational analysis of condensed or fragmented nuclear morphology in various cells treated with *F. cucullata* extract or its subcomponents. Data represent mean ± S.E.M. (standard error of the mean), n = 3. **p<0.01; ***p<0.001; NS, no significant difference compared to the dimethylsulfoxide-treated group.

To determine whether the cytotoxicity of *F. cucullata* and usnic acid was due to the induction of apoptosis, cells treated with a lethal concentration of *F. cucullata* (50 µg/mL) or usnic acid (100 µM) were stained with Hoechst 33258 and their nuclear morphology was observed. As shown in Figure 2A, condensed nuclear morphology was seen in AGS cells treated with either *F. cucullata* extract or usnic acid. In Figure 2B, quantifications of cell numbers in various cell lines are shown. Interestingly, induction of nuclear condensation was significantly increased in treated cancer cells but was not seen in the non-cancer cell line MDCK, suggesting that *F. cucullata* and usnic acid are selectively cytotoxic to cancer cells through the induction of apoptosis.

**Figure 3:**
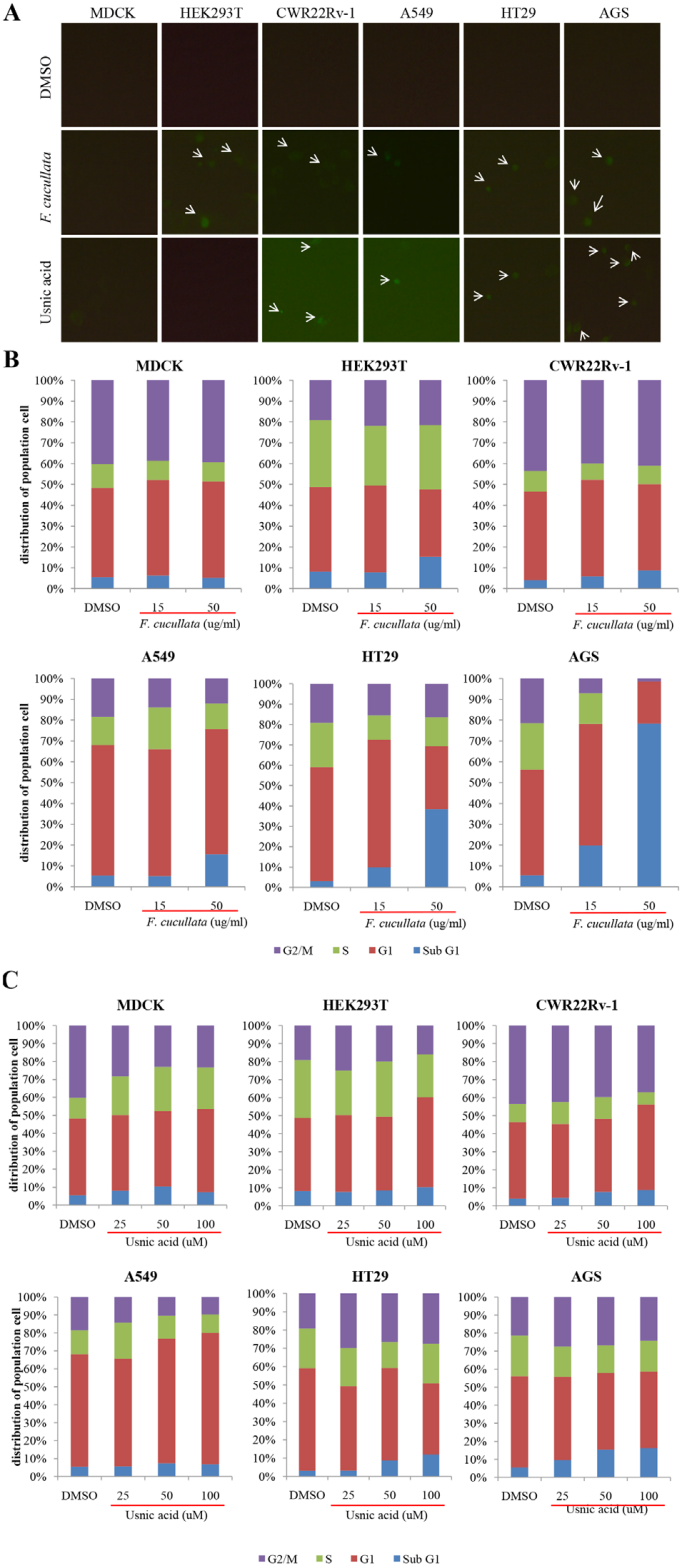
Induction of Annexin V positivity and accumulation of sub G1 population on human cancer cells by the acetone extract of *F. cucullata* and usnic acid in lethal concentrations. (A) FITC-Annexin V staining of cells treated with the *F. cucullata* extract or usnic acid. Arrows indicate cells showing FITC positivity. (B–C) Flow cytometric analysis of cell-cycle distributions after *F. cucullata* extract (B) or usnic acid (C) treatment and graphical representation of the results. Representative images or results are shown from three independent experiments.

To confirm this, we stained the cells with FITC-Annexin V to detect exposure of phosphatidylserine on the outer plasma membrane, which is a characteristic finding in cells undergoing apoptosis. As shown in Figure 3A, cancer cells treated with either *F. cucullata* extract or usnic acid showed FITC positivity. To determine the percentage of apoptotic cells, flow cytometric analysis of cells stained with propidium iodide was performed. As shown in Figures 3B and 3C, the population of cells in the sub G1 phase increased following 24 hr treatment in cancer cells. This quantification analysis revealed that the induction of apoptosis by the *F. cucullata* extract was dose-dependent except in MDCK cells, with significant increases seen in AGS, HT29, and A549 cells (Fig. 3B). However, as shown in Figure 3C, the effectiveness of usnic acid in increasing the number of apoptotic cells was significantly lower than that of the *F. cucullata* extract. These findings again suggest that unidentified subcomponent(s) of *F. cucullata* may potentiate the effects of usnic acid although usnic acid plays a major role in inducing apoptosis in various cancer cells.

**Figure 4:**
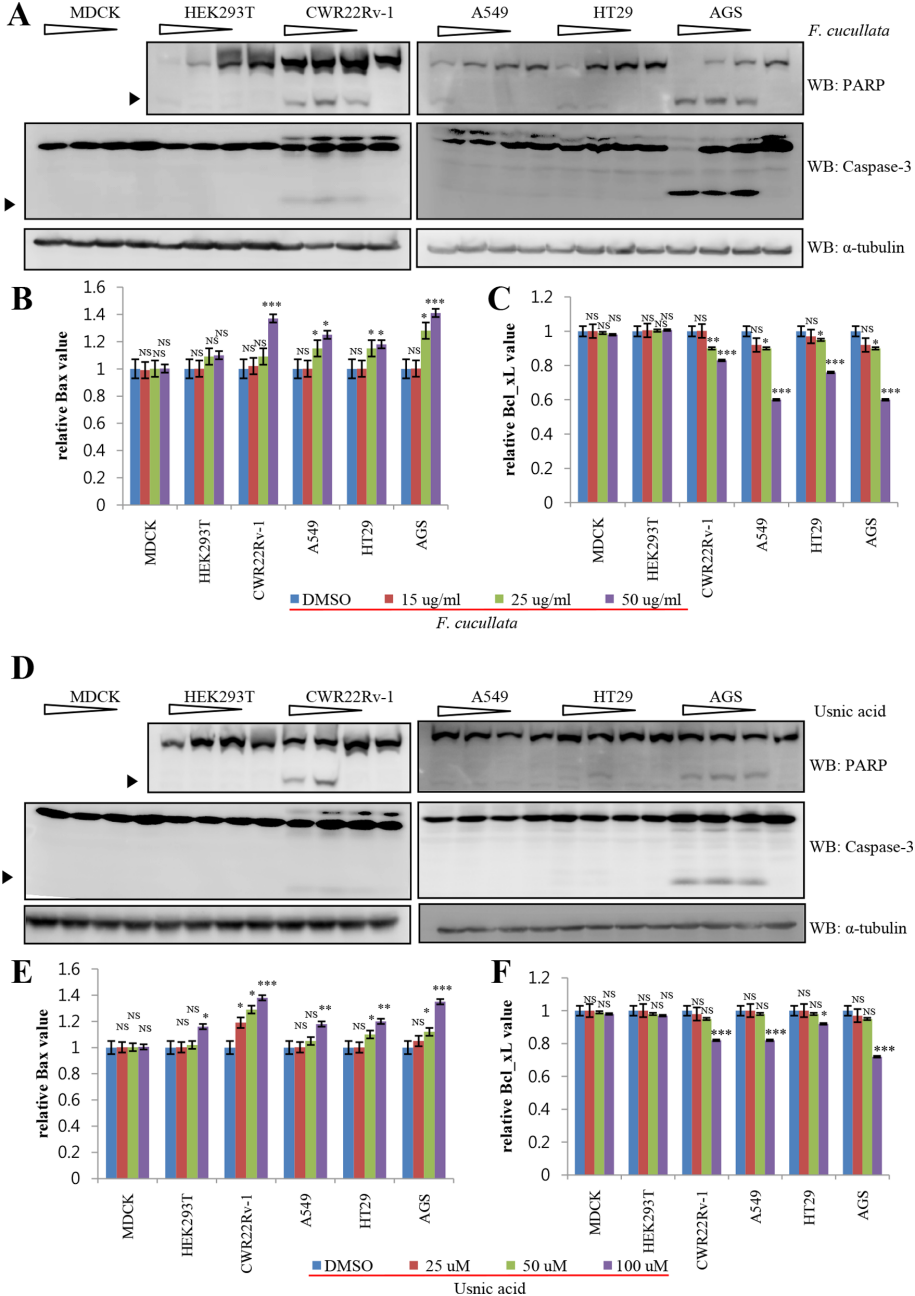
Activation of apoptosis pathway on human cancer cells by the acetone extract of *F. cucullata* and usnic acid in lethal concentrations. (A and D) Western blot analysis of poly (ADP-ribose) polymerase (PARP) and caspase-3 in cells treated with the *F. cucullata* (A) or usnic acid (D). Arrowheads indicate cleaved fragments of each protein. (B–C and E–F) Quantificational analysis of Bax (B and E) and Bcl-xL (C and F) protein expression levels in cells treated with the F. cucullata or usnic acid, respectively. Data represent mean ± S.E.M. (standard error of the mean). *p<0.05; **p<0.01; ***p<0.001; NS, no significant difference compared to the dimethylsulfoxide-treated group.

To further confirm changes in the level of apoptotic proteins, we performed western blot analysis for poly (ADP-ribose) polymerase (PARP), caspase-3, Bax, and Bcl-xL. As shown in Figures 4A and 4D, extract and usnic acid treatment increased the levels of cleaved PARP and cleaved caspase-3 in CWR22Rv-1, AGS, HT29, and A549 cells. In addition, the level of the pro-apoptotic protein, Bax, was significantly increased in these treated cells, but not in MDCK and HEK293T cells (Figs. 4B and 4E). By contrast, the level of the anti-apoptotic protein, Bcl-xL, was significantly decreased only in treated cancer cells (Figs. 4C and 4F). Consistently, *F. cucullata* was more effective in inducing apoptosis than usnic acid and was more effective in cancer cells than non-cancer cell. Taken together, the results demonstrate that lethal concentrations of the acetone extract of *F. cucullata* and the subcomponent of usnic acid induce apoptosis of cancer cells.

### Sub-lethal concentrations of *Flavocetraria cucullata* and usnic acid inhibit tumorigenicity and motility of cancer cells

**Figure 5:**
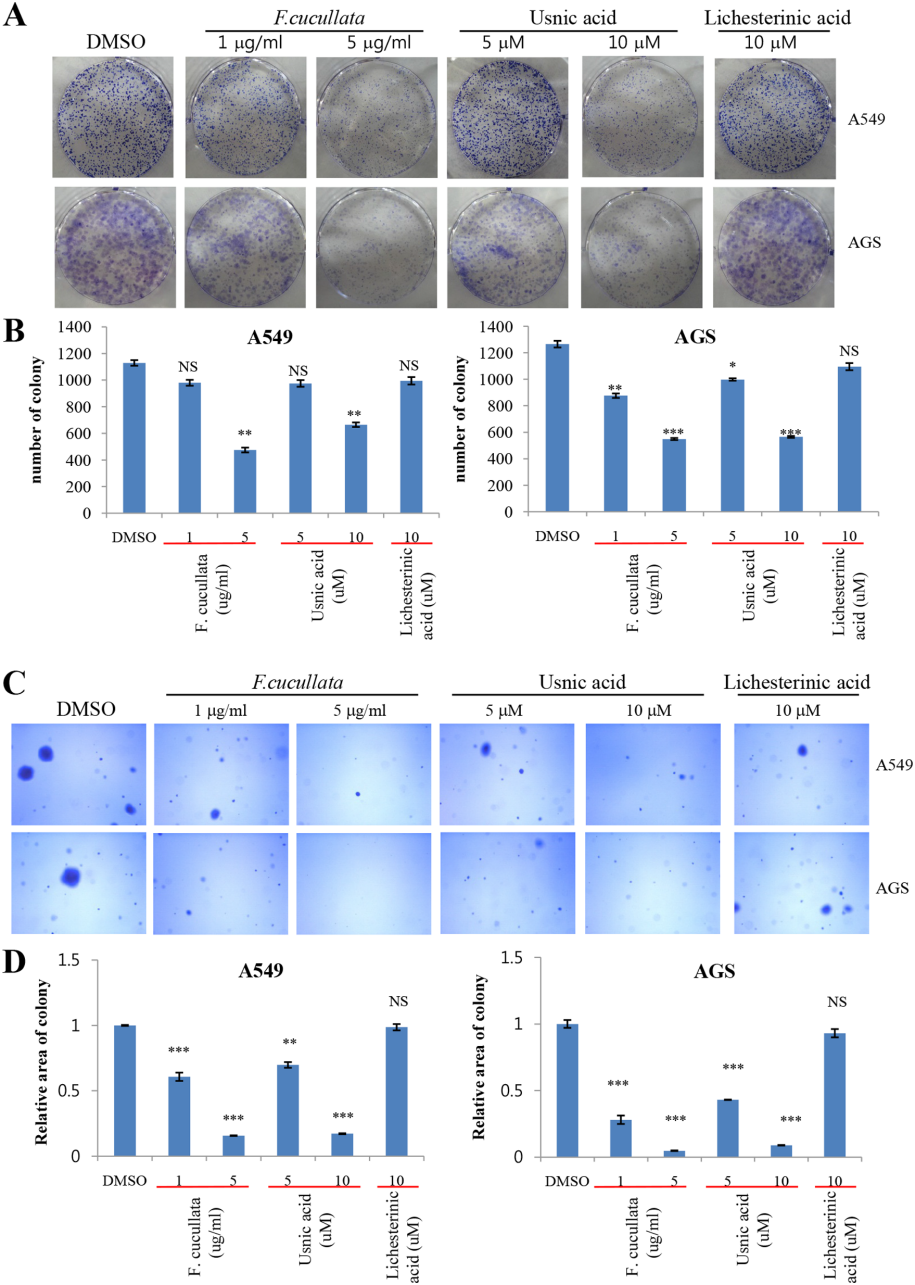
Inhibition of anchorage-independent growth of A549 and AGS cancer cells by the acetone extract of *F. cucullata* and usnic acid in sub-lethal concentrations. (A–B) Clonogenic assay of A549 and AGS cells treated with the *F. cucullata* extract, usnic acid, or lichesterinic acid (A) and quantificational analysis of colony number in each group (B). (C–D) Soft agar colony-formation assay of A549 and AGS cells treated with *F. cucullata* extract, usnic acid, or lichesterinic acid (C) and quantificational analysis of percent colony area in each group (D). Representative images are shown from three independent experiments. Data represent mean ± S.E.M. (standard error of the mean), n = 3. *p<0.05; **p<0.01; ***p<0.001; NS, no significant difference compared to the dimethylsulfoxide-treated group.

**Figure 6:**
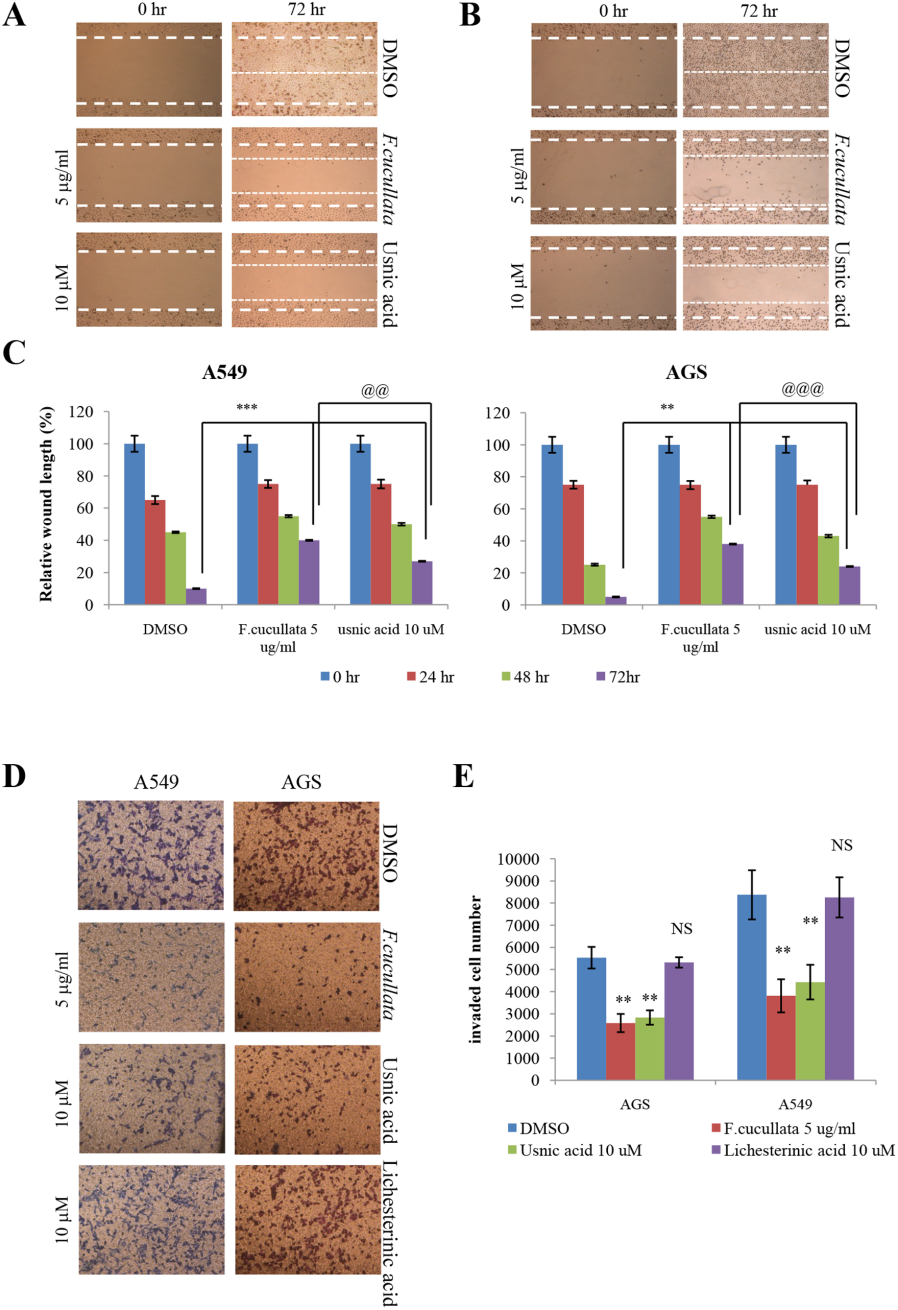
Inhibition of the motility of A549 and AGS cancer cells by the acetone extract of *F. cucullata* and usnic acid in sub-lethal concentrations. (A–C) Migration assay of A549 (A) and AGS (B) cells treated with the *F. cucullata* extract or usnic acid, and quantificational analysis of wound length in each group (C). (D–E) Invasion assay of A549 and AGS cells treated with the *F. cucullata* extract, usnic acid, or lichesterinic acid (D), and quantificational analysis of invaded cell numbers in each group (E). Representative images are shown from three independent experiments. Data represent mean ± S.E.M. (standard error of the mean), n = 3. **p<0.01; ***p<0.001; NS, no significant difference when compared to the dimethylsulfoxide-treated group in each cell lines. @@p<0.01; @@@p<0.001 when compared to the indicated group.

To further explore the anti-cancer activity of the *F. cucullata* extract and usnic acid, we used one-tenth of the lethal concentrations of these compounds which did not showing the cytotoxicity (sub-lethal concentration) and tested the *in* *vitro* tumorigenicity and motility of A549 and AGS cells. A clonogenic assay of these cells at sub-lethal concentrations of the extract (5 µg/mL) or usnic acid (10 µM) showed a significant decrease in the number of colonies, indicating that cell proliferation was inhibited at these concentrations (Figs. 5A and 5B). Interestingly, treatment by 10 µg/mL extract or 15 µM usnic acid completely inhibited the formation of colonies in both cell types (data not shown). By contrast, lichesterinic acid showed no inhibitory effect on cell proliferation. A soft agar colony-formation assay was performed to test whether sub-lethal concentrations of *F. cucullata* and usnic acid inhibited anchorage-independent growth of A549 and AGS cells. As shown in Figures 5C and 5D, colony formation of A549 and AGS cells on soft agar was significantly decreased by treatment with the *F. cucullata* extract or usnic acid in a dose-dependent manner, while lichesterinic acid did not affect anchorage-independent growth. These results demonstrate that both the *F. cucullata* extract and usnic acid have anti-tumorigenic activity at sub-lethal concentrations. A wound-healing assay and invasion assay were further performed to test whether *F. cucullata* and usnic acid affect migration and invasion, respectively, of cancer cells at sub-lethal concentrations. In Figures 6A–C, *F*. *cucullata* extract and usnic acid treatment significantly inhibited the migration of A549 and AGS cells. As shown in Figures 6D–E, the *F. cucullata* extract and usnic acid also inhibited the invasion of A549 and AGS cells, while no such inhibition of invasion was detected in lichesterinic acid-treated cancer cells. The results clearly show that *F. cucullata* and usnic acid inhibit cancer cell motility and decrease tumorigenicity of cancer cells at sub-lethal concentrations.

**Figure 7:**
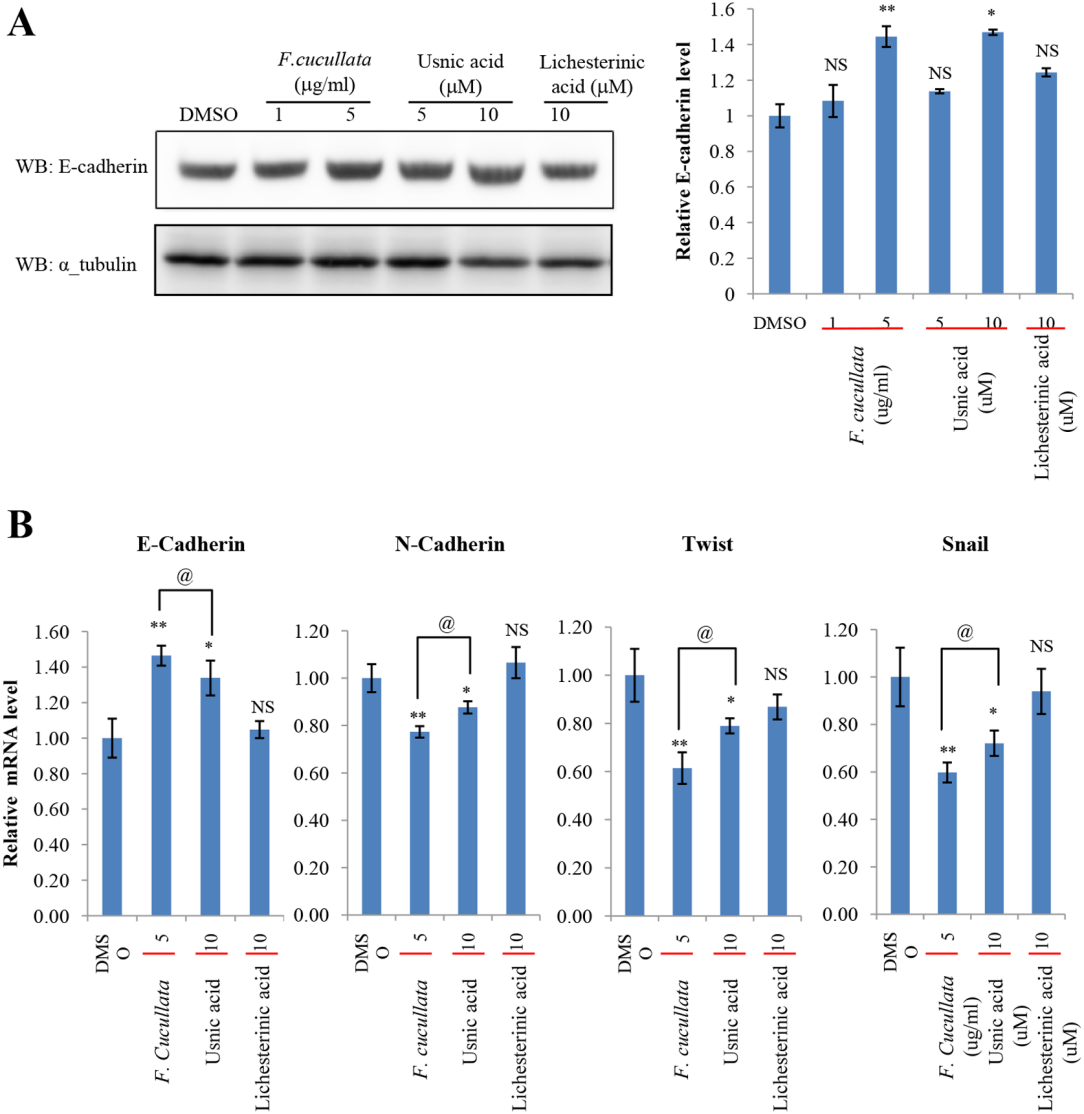
Suppression of epithelial-mesenchymal transition (EMT) by the acetone extract of *F. cucullata* and usnic acid in sub-lethal concentrations. (A) E-cadherin level in A549 cells treated with the *F. cucullata* extract, usnic acid, or lichesterinic acid for 48 hr, and quantificational analysis of E-cadherin band in each group. Values were obtained by measuring the intensity of E-cadherin band normalized to α-tubulin. (B) Quantitative analysis of the mRNA level of EMT markers in A549 cells treated with the *F. cucullata* extract, usnic acid, or lichesterinic acid for 48 hr. Data represent mean ± S.E.M. (standard error of the mean), n = 3. *p<0.05; **p<0.01; NS, no significant difference when compared to the dimethylsulfoxide-treated group; ^@^p<0.05 when compared to the indicated group.

**Figure 8:**
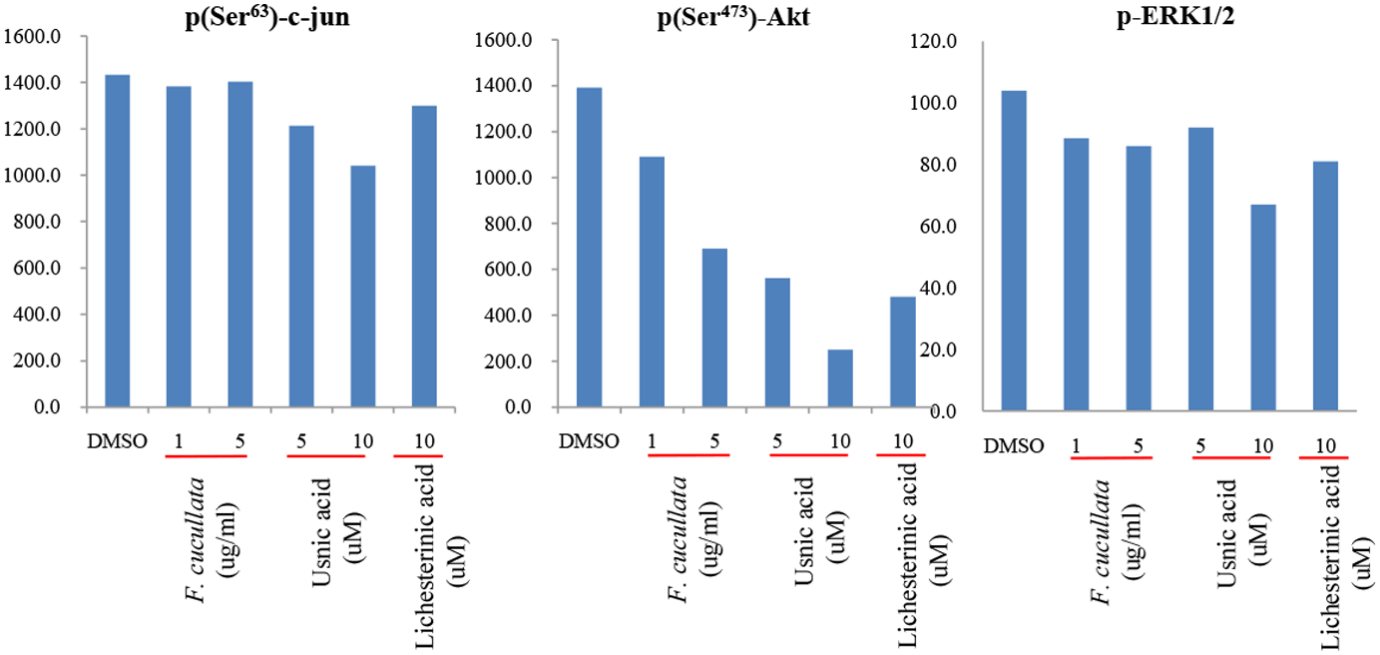
Reduction of phosphor-Akt level by the acetone extract of *F. cucullata* and usnic acid in sub-lethal concentrations. Phosphoprotein analysis for p(Ser^63^)-c-jun, p(Ser^473^)-Akt, and p-(Thr^202^/Tyr^204^, Thr^185^/Tyr^187^)-ERK1/2 in A549 cells treated with the *F. cucullata* extract, usnic acid, or lichesterinic acid.

As *F. cucullata* and usnic acid were found to significantly decrease cancer cell motility and tumorigenicity, we then investigated whether epithelial-mesenchymal transition (EMT) plays a role in mediating these effects. The expression level of E-cadherin was analyzed in A549 cells treated with a sub-lethal concentration of *F. cucullata*, usnic acid, or lichesterinic acid. The data showed that the acetone extract of *F. cucullata* and usnic acid significantly increased the protein level of E-cadherin (Fig. 7A). Consistently, the expression level of E-cadherin mRNA was also increased in these cells, while mRNA levels of N-cadherin, Twist, and Snail were decreased in these cells. These findings indicate that *F. cucullata* and usnic acid can inhibit EMT (Fig. 7B). Lichesterinic acid treatment induced no significant changes in the levels of these EMT markers. In addition, changes in the phosphorylation levels of c-jun, Akt, and ERK1/2 were analyzed in A549 cells treated with a sub-lethal concentration of *F. cucullata*, usnic acid, or lichesterinic acid. As shown in Figure 8, the level of p-(Ser^473^)-Akt was dramatically decreased by both *F. cucullata* and usnic acid treatment in a dose-dependent manner while the levels of p-(Ser^63^)-c-jun and p-(Thr^202^/Tyr^204^, Thr^185^/Tyr^187^)-ERK1/2 were only marginally affected by usnic acid. These results suggest that sub-lethal concentrations *F. cucullata* extract and usnic acid can exert anti-cancer effects possibly through inhibiting EMT and Akt signaling.

**Figure 9:**
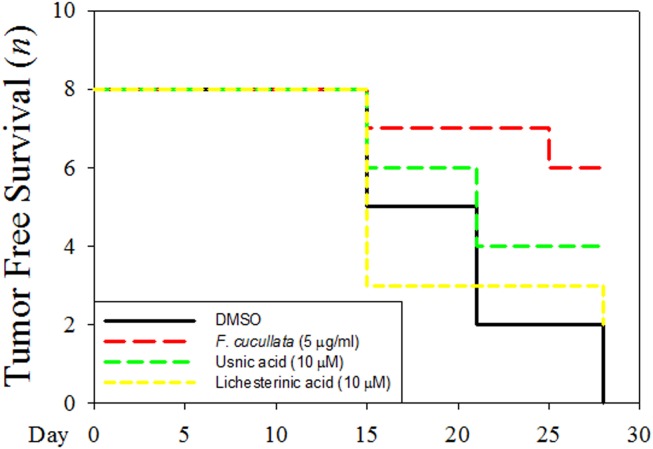
Inhibition of *in* *vivo* tumorigenicity of A549 cancer cells pretreated by the acetone extract of F. cucullata and usnic acid in sub-lethal concentrations. A549 cells were pretreated with indicated concentration of *F. cucullata*, usnic acid, or lichesterinic acid before subcutaneous injection into Balb/c nude mouse (n = 8) and tumor free survival number in each group during 4 weeks were measured.

To further support the anti-cancer effects of *F. cucullata* extract and usnic acid at sub-lethal concentration, we performed the *in* *vivo* tumorigenicity assay. As shown in Figure 9 and Figure S1 in File S1, tumor free survival number in *F. cucullata* pretreated group is highest (tumor free in six out of eight mouse) among DMSO (zero out of eight), usnic acid (four out of eight), or lichesterinic acid (two out of eight) pretreated group. Interestingly, when comparing the *F. cucullata* and usnic acid pretreated group, it is worth noting that tumor free survival number was less in the usnic acid treated group than that of the *F. cucullata* treated group. Taken together, these results demonstrate that *F. cucullata* extract and usnic acid have *in* *vitro* and *in* *vivo* anti-cancer effects at sub-lethal concentration.

## Discussion

Lichen species living at high elevation are occasionally exposed to strong sunlight, including UV radiation, and to severe fluctuations in temperature during their growing seasons. Severe environmental stresses can stimulate the production of chemical protectants for lichens to cope with stress-induced damage, e.g., many lichen pigments are well known to protect lichen thalli from strong UV radiation [5]. Furthermore, several antioxidant compounds have been extracted from lichens growing in harsh environmental conditions [21]. In this study, we investigated the anti-cancer activity of Romanian Carpathian lichens against several cancer cell lines. Among the 17 lichens species tested in this study, *F. cucullata* exhibited the most potent cytotoxicity against HT29 and AGS cancer cell lines. To the best of our knowledge, this is the first report at the molecular level on the anti-cancer activity of lethal and sub-lethal doses of *F. cucullata* and usnic acid against several cancer cell lines. We found the following: 1) the acetone extract of *F. cucullata* and its subcomponent, usnic acid, exert selective cytotoxicity on cancer cells through inducing apoptosis at lethal concentrations; 2) the extract of *F. cucullata* and usnic acid inhibit tumorigenesis and motility of cancer cells at sub-lethal concentrations; 3) the extract of *F. cucullata* and usnic acid suppress EMT and inhibit Akt phosphorylation; and 4) the anti-cancer activity of the extract is more potent than that of usnic acid alone.

Lichen is easily recognized on moss or soil due to its yellow color and curled thalli, and the yellowish thalli indicate that lichen contains usnic acid. HPLC analysis of the acetone extract of thalli confirmed usnic acid as the major compound of the extract, which has been shown to possess many biological activities [5]. Salazinic acid, squamatic acid, baeomycesic acid, d-protolichesterinic acid, and lichesterinic acid have also been detected as additional compounds in thalli. Usnic acid has been previously shown to induce apoptosis of various cancer cell lines, such as human ovarian carcinoma A2780, human colon adenocarcinoma HT29, human breast adenocarcinoma MCF-7, human cervix adenocarcinoma HeLa, human promyelocytic leukemia, human T-cells lymphocyte leukemia, Jurkat, and human breast adenocarcinoma SK-BR-3 cells, at lethal doses through a caspase-dependent pathway by activating caspase-3 [5]–[10], [22], [23]. More specifically, Singh et al. [24] reported that usnic acid inhibits the growth of A549 human lung carcinoma cells and induces cell-cycle arrest and apoptosis. In addition, recent reports showed that the anti-cancer activity of lethal doses of protolichesterinic acid in several cancer cell lines acts via induction of apoptosis through inhibition of the expression of protein Hsp70 in prostate cancer cell lines and activation of caspase-3 in HeLa cell lines [10], [11]. Salazinic acid was also reported to have cytotoxic effects on several cancer cell lines, such as FemX (human melanoma) and LS174 (human colon carcinoma) cells [25]. In this study, the acetone extract of lichen thalli and usnic acid at lethal doses showed selective cytotoxicity to several cancer cell lines by inducing apoptosis; however, the acetone extract showed more potent cytotoxic effects on the tested cancer cell lines than usnic acid alone. This finding suggests that additional compounds of the lichen such as protolichesterinic and salazinic acid may contribute to the cytotoxic effects of the acetone extract.

There are many examples of compounds derived from natural products possessing inhibitory activity on invasion and metastasis [26]–[28]. However, nothing is known from lichen species. Instead, strong wound closure effect was observed in HaCaT cells (human keratinocyte) in the presence of sub-lethal doses of usnic acid and gyrophoric acid suggesting its possible role in tissue regeneration [29]. Sub-lethal doses of the acetone extract of *F. cucullata* and usnic acid inhibited tumorigenesis and motility of cancer cell lines. Investigation of the expression of intracellular signaling markers involved in the metastatic pathway showed that the extract suppressed the expression of EMT markers and inhibited phosphorylation of Akt. Similar to our findings of the synergistic effects with several compounds (e.g., salazinic acid) of the extract on apoptosis at lethal doses, the acetone extract also exerted more potent inhibition of markers of the metastatic pathway compared to usnic acid alone. Recently, Song et al. reported that usnic acid at sub-lethal doses suppressed the angiogenic potential in a mouse xenograft tumor model and significantly inhibited endothelial cell proliferation, migration and tube formation by inhibiting pAKT and pERK1/2 levels in endothelial cells [30]. Consistently, our results showed that usnic acid also decreased pAKT and pERK1/2 level in A549 cells (Fig. 8). However, the potent effects of the acetone extract of *F. cucullata* was not observed in decreasing pAKT level suggesting that inhibition of EMT is more prominent by the acetone extract of *F. cucullata* and act a crucial role in the inhibition of tumorigenesis and motility of cancer cells.

In this study, our present results suggest that usnic acid plays a major role in the regulation of apoptosis, tumorigenesis, and motility of cancer cells, while additional subcomponents such as salazinic acid synergistically exert anti-cancer activity against the cancer cells alongside usnic acid. In this regards, our findings add novel insight into an anti-cancer activity of lichen species. First, as the acetone extract of lichen thalli and usnic acid showed either selective cytotoxicity or inhibitory activity on tumorigenesis and motility of cancer cells at lethal or sub-lethal doses, respectively, selective cytotoxicity to cancer cells might be obtained via dose-adjustment of the acetone extract of lichen thalli and suggest that this novel anti-cancer chemotherapeutic agent can be used as a selective and effective agent against various cancers. Second, as the acetone extract of lichen thalli showed more potent cytotoxic effects on the tested cancer cells than usnic acid alone, whole acetone extract of lichen thalli may be more effective as a new therapeutic agent than usnic acid alone in treating cancer patients. Therefore, these findings support a rationale for combined using of additional compounds of the lichen as well as usnic acid to improve responses to anti-cancer therapy in patients resistant to routine cancer chemotherapy. Further study is required to reveal additional molecular mechanisms underlying the anti-cancer activity of the lichen species and their secondary metabolites.

## Supporting Information

File S1 Includes Figure S1, Tables S1–S3. **Figure S1.** In vivo tumorigenicity assay using A549 cells pretreated with DMSO, acetone extract of *F. cucullata*, usnic acid, or lichesterinic acid. A549 cells were pretreated with DMSO, *F. cucullata* (5 µg/mL), Usnic acid (10 µM) or Lichesterinic acid (10 µM) before injection. Pictures were taken after 28 days of subcutaneous cell injections. **Table S1.** IC_50_ values of the acetone extract from thalli of the Romania lichens. **Table S2.** Mass spectrometry of the *Flavocetraria cucullata* acetone extract. **Table S3.** IC_50_ values of the acetone extract of *F. cucullata* and usnic acid, lichesterinic acid, on various cancer cells.
(PDF)
