# Expression of Concern: Lichen Secondary Metabolites in *Flavocetraria cucullata* Exhibit Anti-Cancer Effects on Human Cancer Cells through the Induction of Apoptosis and Suppression of Tumorigenic Potentials

**DOI:** 10.1371/journal.pone.0282452

**Published:** 2023-02-24

**Authors:** The PLOS ONE Editors

After this article [1] was published, concerns were raised about similarities between some panels in Figs 3A and 6D. Specifically:

- In Fig 3A, the DMSO, HEK293T panel appears duplicated in the Usnic acid, HEK293T panel when levels are adjusted.
- In Fig 3A, the *F*. *cucullata*, HT29 panel appears duplicated in the Usnic acid, HT29 panel.
- In Fig 6D, the top right of the Lichesterinic acid, AGS panel overlaps with the bottom left of the DMSO, AGS panel.

In response to these concerns, the corresponding author stated that the Usnic acid, HEK293T panel in Fig 3A was inadvertently duplicated from the DMSO, HEK293T panel. The corresponding author also stated that the *F*. *cucullata*, HT29 and Usnic acid, HT29 panels in Fig 3A were inadvertently duplicated but that they are not able to verify if either of these panels are correct. An updated version of Fig 3A is provided here where the Usnic acid, HEK293T panel has been replaced by an image from the time of the original experiment, and where the *F*. *cucullata*, HT29 and Usnic acid, HT29 panels have been removed. The corresponding author also stated that the DMSO, AGS panel in Fig 6D was inadvertently duplicated from the Lichesterinic acid, AGS panel. An updated version of Fig 6D is provided here where the DMSO, AGS panel has been replaced by an image from the time of the original experiment.

The corresponding author provided original data underlying the HEK293T and A549 panels in Fig 3A (S1 File), and the AGS panels in Fig 6D (S2 File), and individual-level data from which the charts in Figs 3B and 3C and 6C were generated (S3 File). The above concern regarding Fig 6D is therefore resolved. The corresponding author stated that although the original data underlying the HEK293T panels in Fig 3A are available, they are not able to confirm which of the DMSO, HEK293T original data files corresponds to the DMSO, HEK293T panel in Fig 3A in [1]. The corresponding author stated that the original data underlying the MDCK, CWR22Rv-1, AGS, and HT29 panels in Fig 3A, all panels in Figs 6A and 6B, the A549 panels in Fig 6D, and the individual-level data from which the chart in Fig 6E was generated are no longer available. The above concerns regarding Fig 3A therefore cannot be fully resolved. The corresponding author stated that the remaining underlying data are also likely no longer available.

In light of unconfirmed original data underlying the DMSO, HEK293T panel in Fig 3A, and unavailability of much of the original data, including underlying the HT29 panels in Fig 3A, the above concerns with Fig 3A cannot be fully resolved. The *PLOS ONE* Editors therefore issue this Expression of Concern due to concerns about the integrity of data reporting in this article.

**Fig 3:**
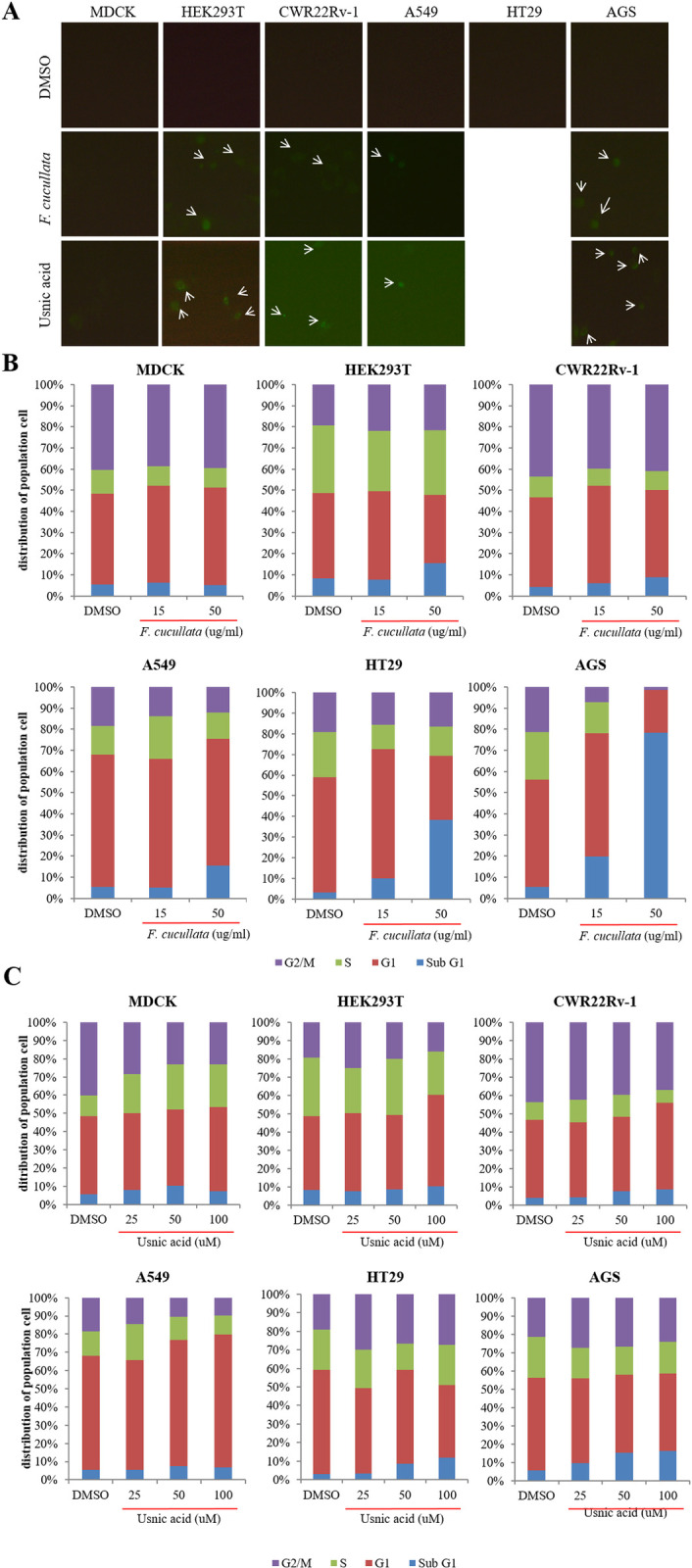
Induction of Annexin V positivity and accumulation of sub G1 population on human cancer cells by the acetone extract of *F*. *cucullata* and usnic acid in lethal concentrations. (A) FITC-Annexin V staining of cells treated with the *F*. *cucullata* extract or usnic acid. Arrows indicate cells showing FITC positivity. (B–C) Flow cytometric analysis of cell-cycle distributions after *F*. *cucullata* extract (B) or usnic acid (C) treatment and graphical representation of the results. Representative images or results are shown from three independent experiments.

**Fig 6:**
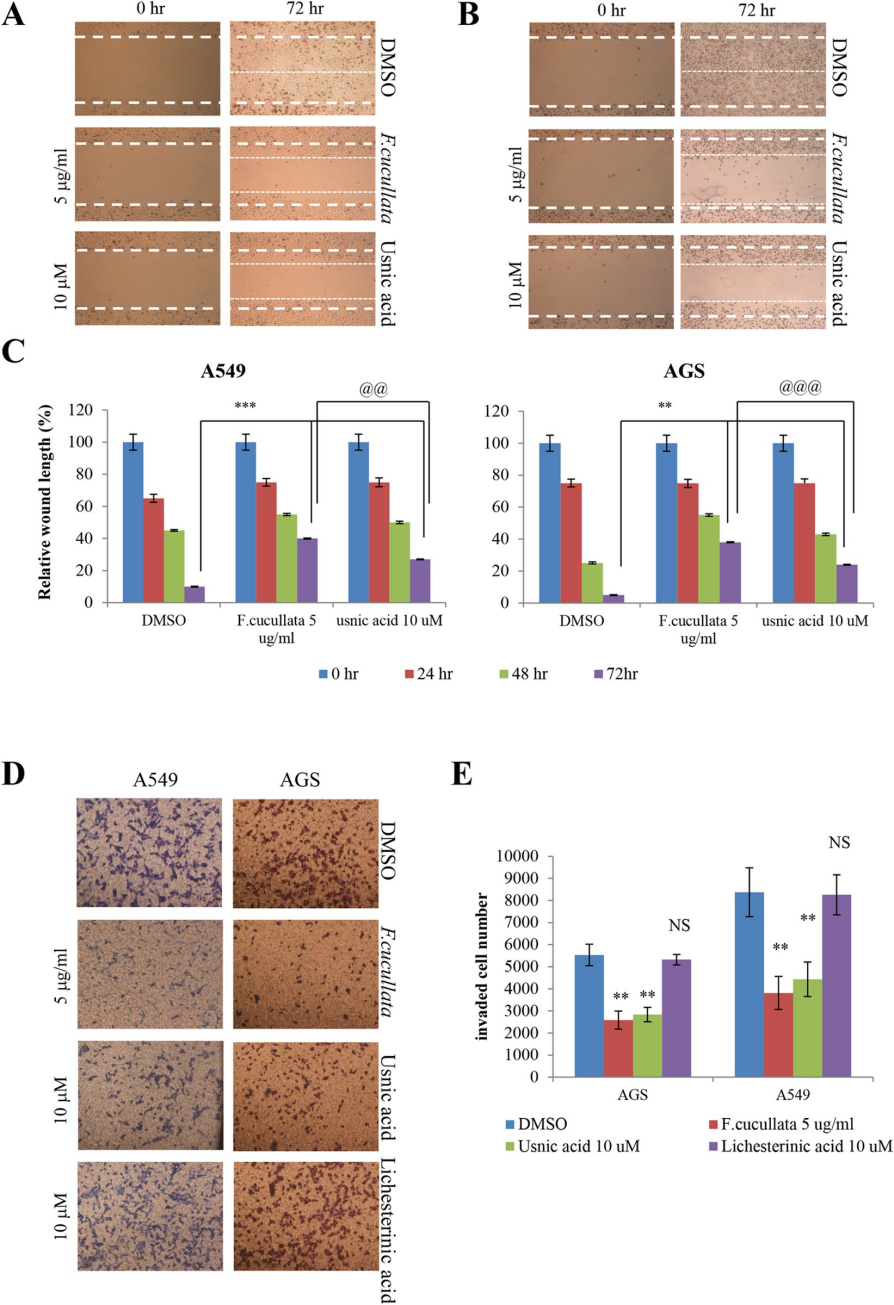
Inhibition of the motility of A549 and AGS cancer cells by the acetone extract of *F*. *cucullata* and usnic acid in sub-lethal concentrations. (A–C) Migration assay of A549 (A) and AGS (B) cells treated with the *F*. *cucullata* extract or usnic acid, and quantificational analysis of wound length in each group (C). (D–E) Invasion assay of A549 and AGS cells treated with the *F*. *cucullata* extract, usnic acid, or lichesterinic acid (D), and quantificational analysis of invaded cell numbers in each group (E). Representative images are shown from three independent experiments. Data represent mean ± S.E.M. (standard error of the mean), n = 3. **p<0.01; ***p<0.001; NS, no significant difference when compared to the dimethylsulfoxide-treated group in each cell lines. ^@@^p<0.01; ^@@@^p<0.001 when compared to the indicated group.

## Supporting information

S1 File Original and replicate underlying data from the time of the original experiments supporting the HEK293T and A549 panels in Fig 3A. (PPTX)

S2 File Original and replicate underlying data from the time of the original experiments supporting the AGS panels in Fig 6D. (PPTX)

S3 File Underlying data supporting the charts in Figs 3B and 3C and 6C. (XLSX)
