# Supplementary material for: Lichen Secondary Metabolites in Flavocetraria cucullata Exhibit Anti-Cancer Effects on Human Cancer Cells through the Induction of Apoptosis and Suppression of Tumorigenic Potentials
Source: PLoS One. 2014 Oct 31;9(10):e111575. doi: 10.1371/journal.pone.0111575 (PMC4216107; doi:10.1371/journal.pone.0111575)
Supplement: File S1 — Includes Figure S1, Tables S1–S3. Figure S1. In vivo tumorigenicity assay using A549 cells pretreated with DMSO, acetone extract of F. cucullata, usnic acid, or lichesterinic acid. A549 cells were pretreated with DMSO, F. cucullata (5 µg/mL), Usnic acid (10 µM) or Lichesterinic acid (10 µM) before injection. Pictures were taken after 28 days of subcutaneous cell injections. Table S1. IC50 values of the acetone extract from thalli of the Romania lichens. Table S2. Mass spectrometry of the Flavocetraria cucullata acetone extract. Table S3. IC50 values of the acetone extract of F. cucullata and usnic acid, lichesterinic acid, on various cancer cells. (PDF) [file pone.0111575.s001.pdf]

Supplementary Material

Figure S1.

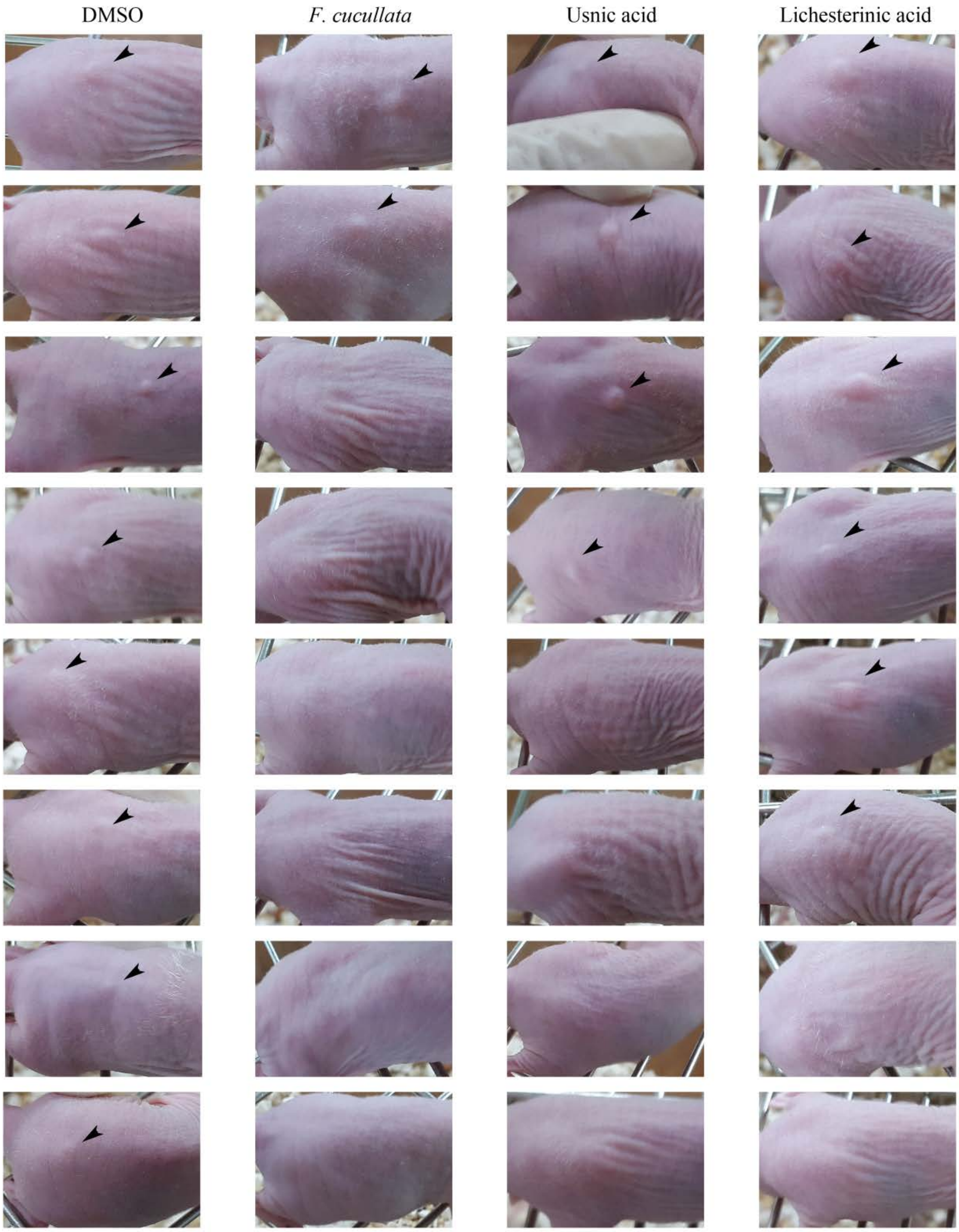

Table S1.

No. collection	**Name of lichen species**	**Main compounds based on TLC analysis results**	**IC_{50} of acetone extract after 2 days (µg/ml)**	
			**HT29**	**AGS**
**RO11002**	***Thamnolia vermicularis (SW) Schaer***	Decarboxythamnolic<br>Thamnolic acid	71.045	87.56
**RO11025**	***Alectoriasa mentosa (Ach) Ach.***	Alectoronic acid<br>Usnic acid	44.3	30.55
**RO11045**	***Flavocetraria nivalis (L.) Karnefelt& A. Thell***	Ergosterol<br>Usnic acid<br>Freidelin	39.02	17.9
**RO11069**	***Stereocaulon alpinum Laurer***	Norstictic acid<br>Lobaric acid<br>Atranorin	46.98	53.8
**RO11073**	***Cladonia mitis Sandst***	Fumarprotocetraric acid<br>Norrangiformic acid<br>Rangiformic acid<br>Psoromic acid<br>Usnic acid	31.63	42.7
**RO11078**	***Cetraria islandica (L.) Ach***	Fumarprotocetraric acid<br>Alloprotolichesterinic acid	73.37	110.7

		Protolichesterinic acid<br>Lichesterinic acid<br>Freidelin		
**RO11079**	***Pseudevernia furfuracea (L.) Zopf***	Physodic acid<br>Chloroatranorin<br>Atranorin	47.14	40.3
**RO11084**	***Alectoria nigricans (Ach) Nyl***	Alectorialic acid	34.2	28.95
**RO11111**	***Bryoria capillaris (Ach.) Brodo& D. Hawksw***	Salazinic acid<br>Psoromic acid<br>Barbatolic acid<br>Atranorin	91.16	41.5
**RO11166**	***Hypogymnia physodes (L.) Nyl***	Physodalic acid<br>Physodic acid<br>Atranorin<br>Chloroatranorin	74.21	40.1
**RO11176**	***Usnea florida (L.) weber ex F.H. Wigg***	Salazinic acid<br>Thamnolic acid<br>Usnic acid<br>Barbatic acid	53.4	65.2
**RO11182**	***Platismatia glauca (L.) W. Culb. & C. Culb.***	Caperatic acid<br>Atranorin	99.537	70.7
**RO11193**	***Peltigera venosa (L.) Hoffm***	Tenuiorin	49.06	36.5
**RO11207**	***Cladonia macrophylla (Schaer.) Stenh***	Psoromic acid	N.D.	N.D.
**RO11208**	***Cetraria ericetorum Opiz***	Fumarprotocetraric acid	39.16	27.8

		Alloprotolichesterinic acid<br>Protolichesterinic acid<br>Lichesterinic acid<br>Freidelin		
**RO11209**	***Evernia divaricata (L.) Ach***	Usnic acid<br>Divaricatic acid	44.65	32.4
**RO11210**	***Flavocetraria cucullata (Bellardi) Karnefelt&A. Thell***	Protolichesterinic acid<br>Usnic acid	18.924	11.6

N.D. indicates that no cytotoxicity was found on cancer cell lines at a drug concentration of 100 μg/mL.

Table S2.

Peak no.	Retention time (min)	% Intensity	Molecular formula	Compounds
1	2.268	2.30 ± 0.1	$C_{18}H_{12}O_{10}$	Salazinic acid
2	2.855	1.06 ± 0.05	$C_{19}H_{18}O_{9}$	Squamatic acid
3	4.646	1.1 ± 0.05	$C_{19}H_{18}O_{8}$	Baeomycesic acid
4	11.327	91.49 ± 0.0025	$C_{18}H_{16}O_{7}$	Usnic acid
5	22.315	2.27 ± 0.1	$C_{19}H_{32}O_{4}$	d-protolichesterinic acid
6	26.595	2.22 ± 0.1	$C_{19}H_{32}O_{4}$	Lichesterinic acid

Table S3.

Cell lines	MDCK	RIE	NIH 3T3	HaCaT	HEK293T	HT29	AGS	A549	CWR22Rv-1
**Acetone extract (μg/ml)**	124.14 ± 2.5	66.5 ± 3.75	84.7±2.5	87.9±1.8	21.82 ± 0.98	18.9 ± 0.25	11.6 ± 0.4	16.65 ± 0.35	32.2 ± 0.75
**usnic acid concentration at IC_{50} in acetone extract (μM)**	328.1	175.8	225.06	233.6	57.6	49.9	30.7	44.01	85.1
**Usnic acid (μM)**	133.04 ± 3.5	126 ± 4.25	164.2±3.7	185.7±4.8	85.3 ± 0.75	95.2 ± 0.85	15.01 ± 0.52	65.3 ± 0.65	24.1 ± 0.63
**Lichesterinic acid (μM)**	N.D.	N.D.	N.D.	N.D.	N.D.	587 ± 1.7	385.9 ± 1.8	N.D.	1015 ± 4.5

N.D. indicates no cytotoxicity was found on cancer cell lines at 100 μg/mL. Data are presented as means ± standard deviation of three independent experiments.

MDCK, Madin-Darby canine kidney; RIE, rat intestinal epithelial; HEK, human embryonic kidney; HT29, human colon cancer cell line; AGS, human gastric cancer cell line; A549, human lung cancer cell line; and CWR22Rv-1, human prostate cancer cell line.
