# Supplementary material for: Expression of Concern: Lichen Secondary Metabolites in Flavocetraria cucullata Exhibit Anti-Cancer Effects on Human Cancer Cells through the Induction of Apoptosis and Suppression of Tumorigenic Potentials
Source: PLoS One. 2023 Feb 24;18(2):e0282452. doi: 10.1371/journal.pone.0282452 (PMC9955601; doi:10.1371/journal.pone.0282452)
Supplement: S1 File — (PPTX) [file pone.0282452.s001.pptx]

## Slide 1
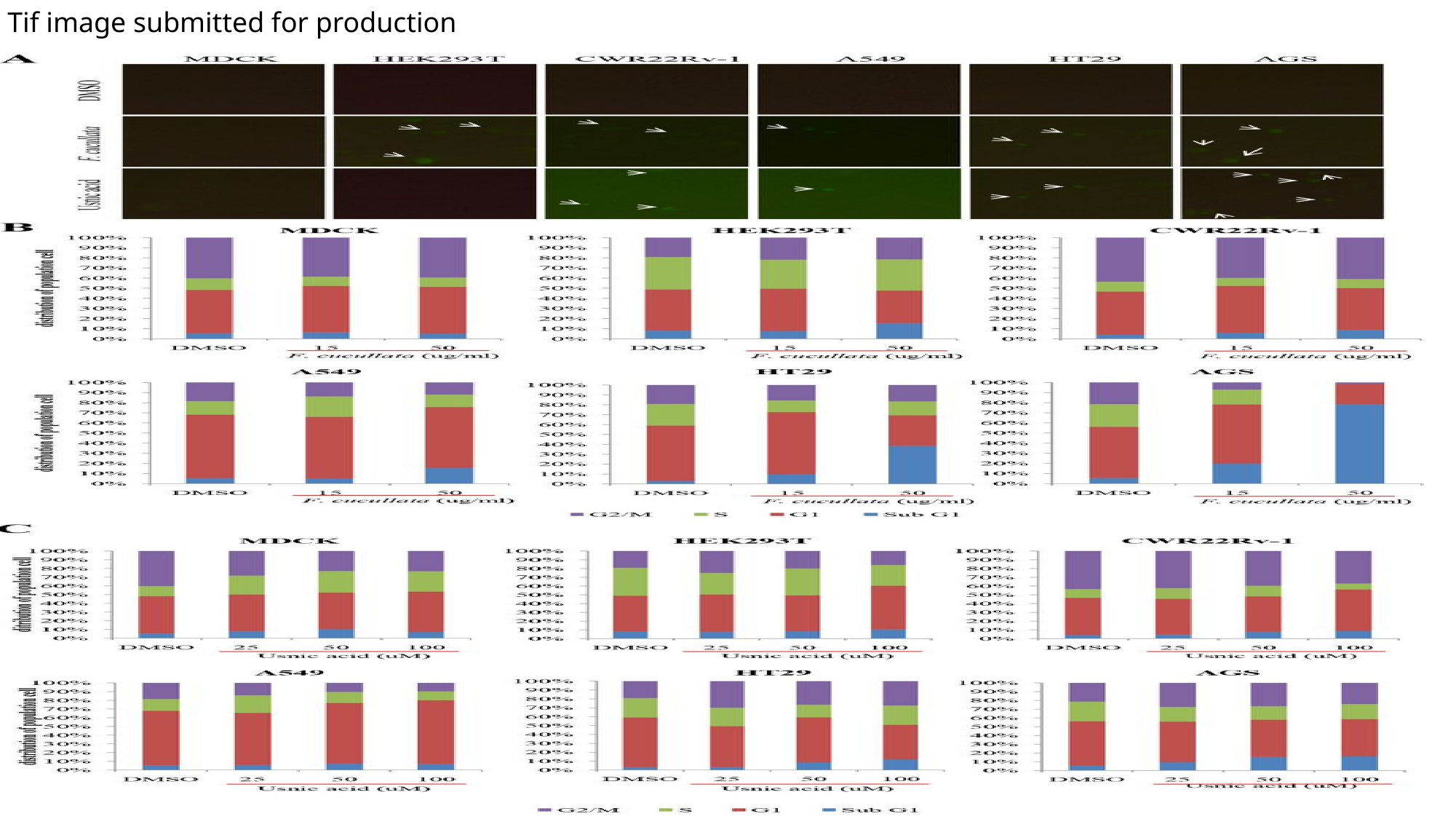

Tif image submitted for production

## Slide 2
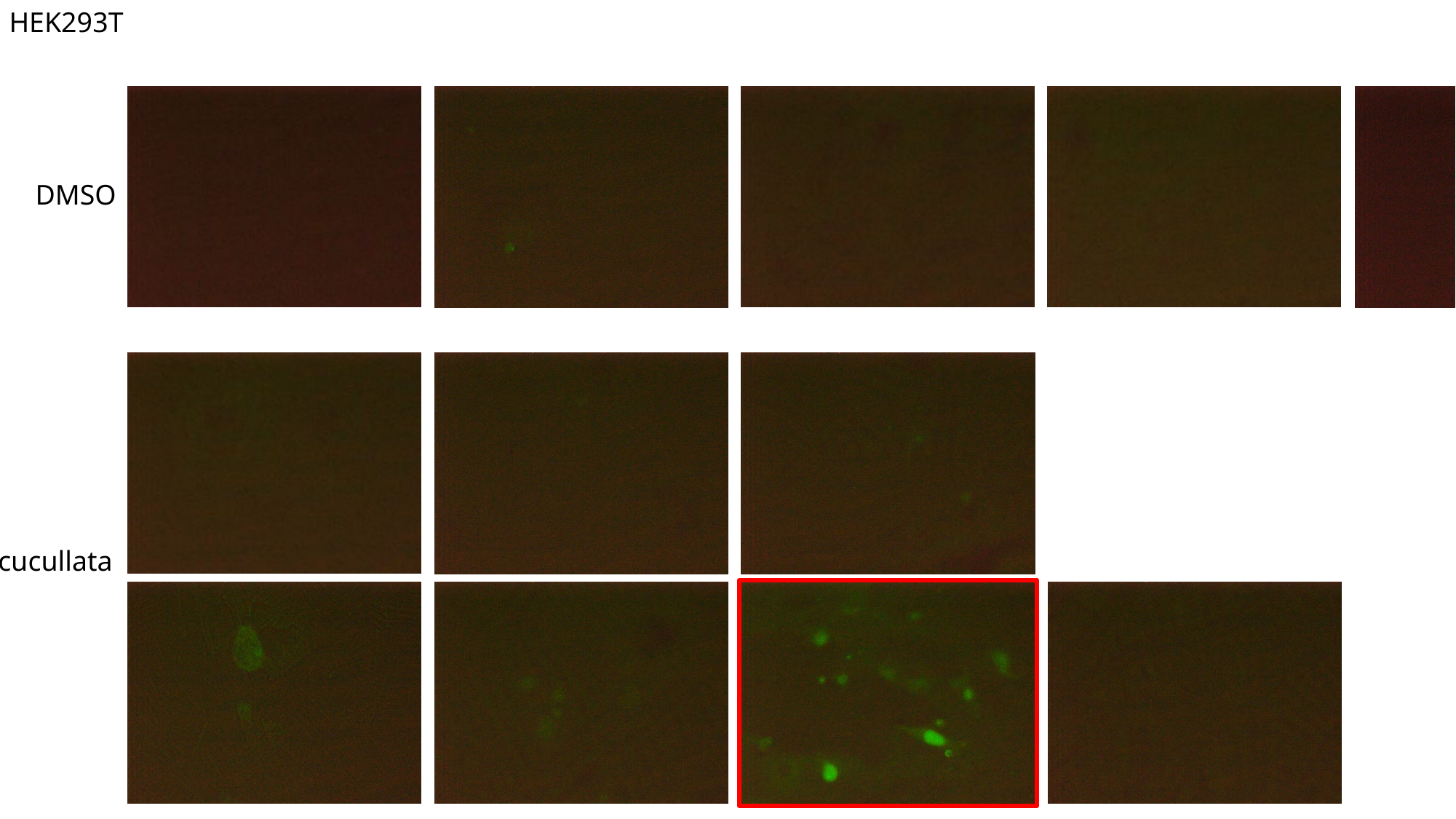

HEK293T
DMSO
F. cucullata
UA

## Slide 3
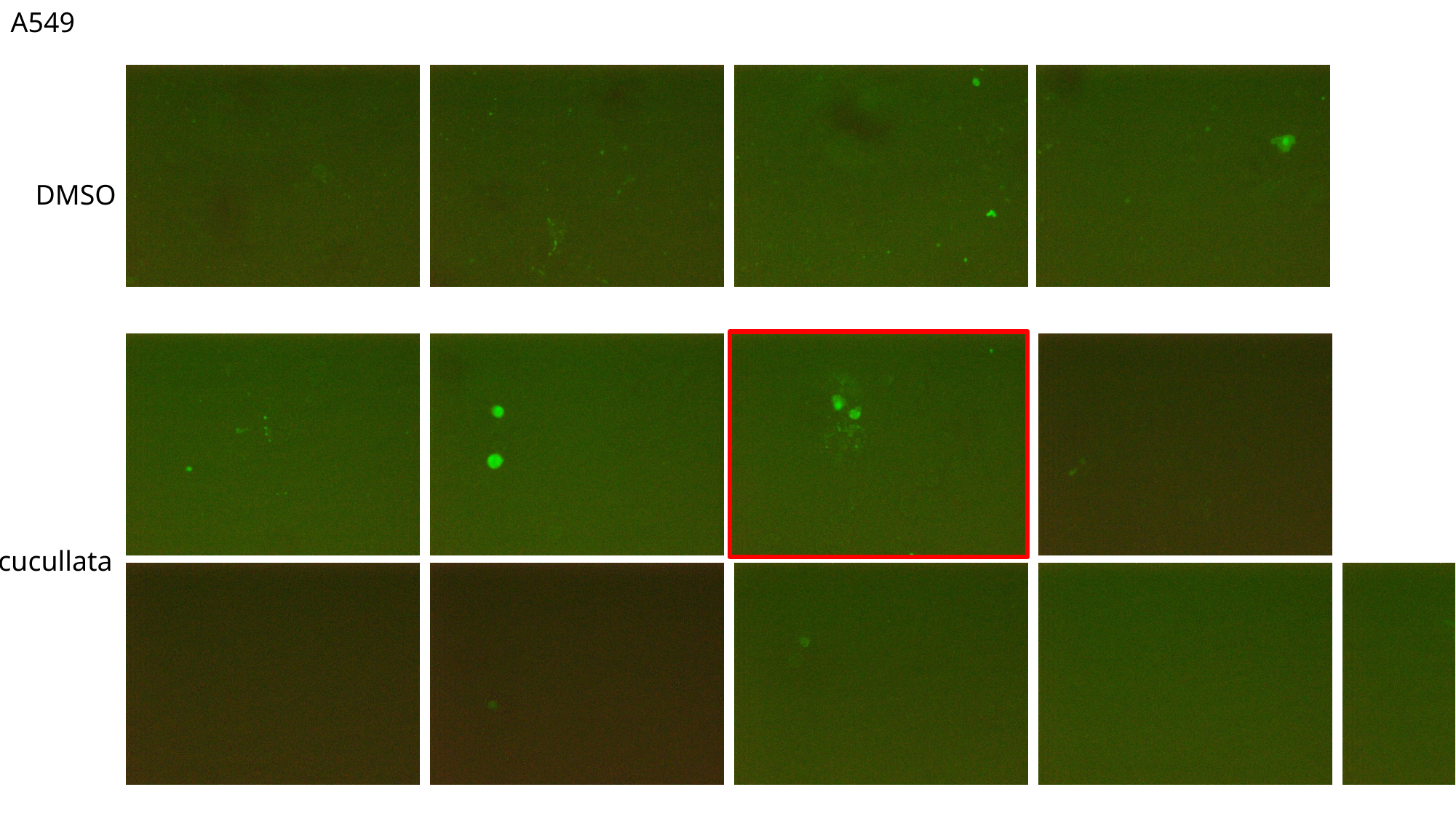

A549
DMSO
F. cucullata
UA
