# Supplementary material for: Expression of Concern: Lichen Secondary Metabolites in Flavocetraria cucullata Exhibit Anti-Cancer Effects on Human Cancer Cells through the Induction of Apoptosis and Suppression of Tumorigenic Potentials
Source: PLoS One. 2023 Feb 24;18(2):e0282452. doi: 10.1371/journal.pone.0282452 (PMC9955601; doi:10.1371/journal.pone.0282452)
Supplement: S2 File — (PPTX) [file pone.0282452.s002.pptx]

## Slide 1
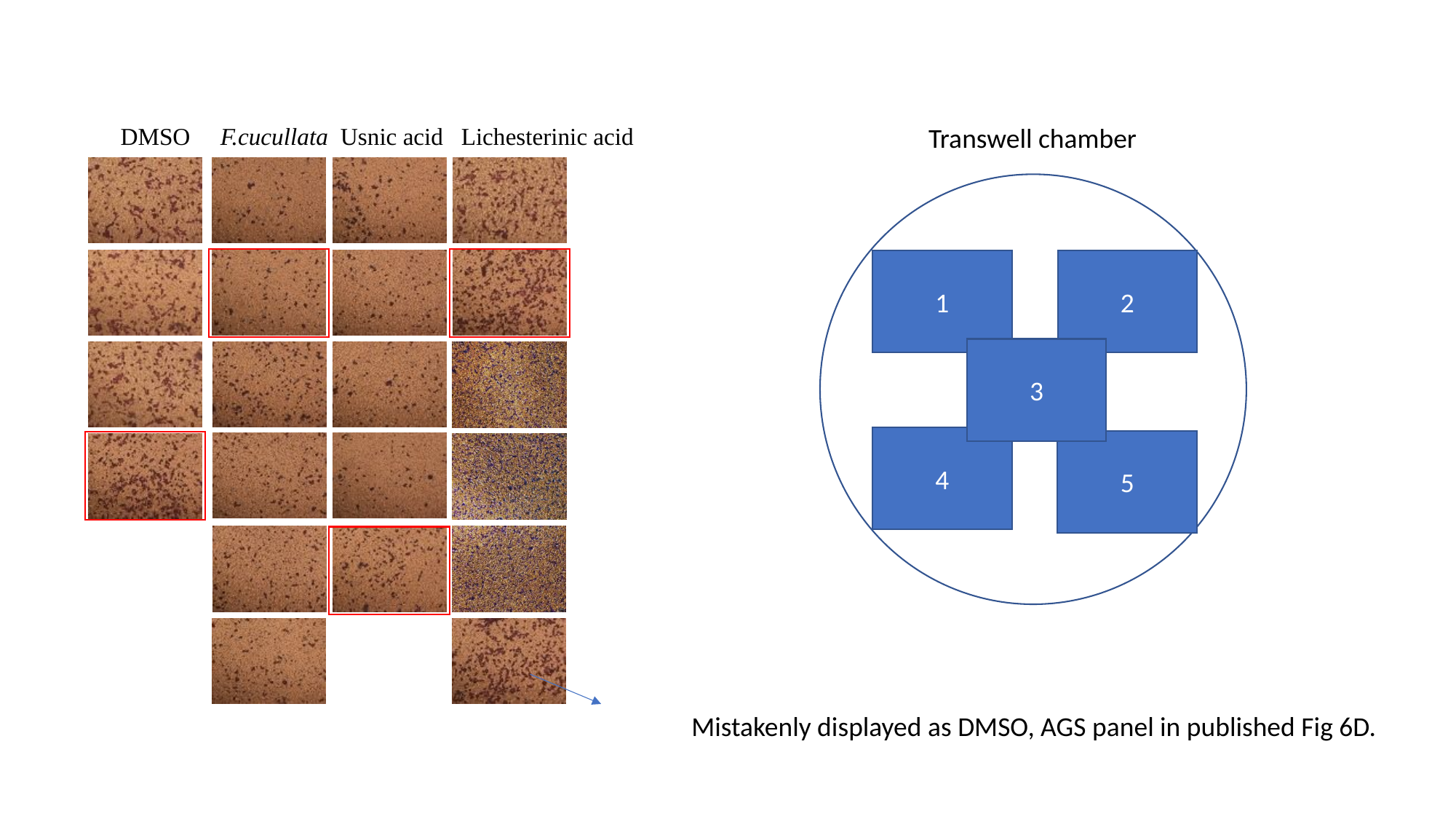

DMSO F.cucullata Usnic acid Lichesterinic acid
Transwell chamber
2
1
3
4
5
Mistakenly displayed as DMSO, AGS panel in published Fig 6D.
